# Recommendations for Mental Health Reforms in Uzbekistan: A Policy Report

**DOI:** 10.5195/cajgh.2020.513

**Published:** 2020-03-31

**Authors:** Akmal Alikhan Aliev, Tatiana Taylor Salisbury

**Affiliations:** 1 Centre for Global Mental Health, Health Service and Population Research Department, Institute of Psychiatry, Psychology and Neuroscience, King's College London, London, UK

**Keywords:** Deinstitutionalization, Policy Report, Uzbekistan

## Abstract

**Introduction:**

There are large differences in the development of mental health systems of the West and the countries of the former Eastern Bloc. The latter is characterized by a more biological approach to mental health and reliance on psychiatric hospitals. In 2018, Uzbekistan authorities showed interest in reforming mental health care of the country. The policy report provides an overview of progress towards the provision of community mental health (CMH) care across Eastern Europe and recommendations for this transition within Uzbekistan.

**Methods:**

A literature search on mental health care in Uzbekistan was conducted to understand its strengths and weaknesses. Progress towards the provision of CMH care across Eastern Europe was assessed using data on the number of psychiatric beds and availability of mental health services in community settings reported within the published literature. Countries identified as making the greatest progress towards CMH care were reviewed in detail to better understand the process of reform assets and barriers.

**Results:**

Mental health care in Uzbekistan is highly institutionalized, underfunded and understaffed. Social care services are poorly developed. However, current leadership has kindled the promise of mental health reform. Georgia, Lithuania and Poland have made the most progress in terms of CMH care availability. However, due to various obstacles such as dual financial burden, high stigma and lack of political will, their programs lack social integration and/or uniform availability and underfunding along with scarcity of mental health specialists are common. On the other hand, research and evaluation, involvement of service users into service planning and cooperation with donors facilitated reform implementation.

**Conclusions:**

Uzbekistan may develop into a modern mental health system and avoid the setbacks encountered by other countries in the region, through careful financial planning, stigma reduction, improving mental health literacy, human resources strategic development and civil society engagement.

The reform of mental health care in Eastern Europe (i.e. the 23 countries of the United Nations Eastern European Group ^1^) has not been as swift as in Western Europe. In the West, mental health care reform began in earnest in the mid-20^th^ century with closures of mental asylums and psychiatric hospitals in response to financial, treatment and societal shifts^2^,^3^ which resulted in major policy shifts favoring community-based mental health care and deinstitutionalization^4^. Community mental health (CMH) is based on the principle that treatment is provided in the least confined environment and aimed at rehabilitating the person for returning to the society^5^. The process of downsizing psychiatric hospitals while expanding mental health services within the community is known as deinstitutionalization^6^.

While deinstitutionalization was commonplace across Western Europe during the end of the 20^th^ A literature search on the Uzbekistan's mental health system published between 1991 (year of Uzbekistan's independence) and April 2019 was conducted across four databases (Medline, Embase, PsychINFO and Web of Science). The search strategy (appendix) was based on a previous review of mental health systems in century, a more biological approach to mental health held across Eastern Europe (EE) supported the institutionalization of mental health care in the region^7^. Although progress towards deinstitutionalization in Europe varies significantly from country to country^8^, across EE it is generally hampered by insufficient numbers of mental health professionals, a significant reliance on inpatient care and a lack of funding^9^.

Uzbekistan, a middle-income country in Central Asia with a population of 32.4 million people^10^, gained independence after the collapse of Soviet Union and has experienced a long period of stagnation with mental health services concentrated primarily in old overcrowded psychiatric institutions with poor sanitary conditions^11^. Recently the country has shown signs of openness for wide-ranging reforms, including in mental health. A revised psychiatric care law is planned to be issued in the near future with a focus on human rights. The presidential decree on mental health published in 2018 aimed at reducing the treatment gap, improving the quality of care in hospitals and increasing the psychiatric workforce^12^. However, the country's new mental health policy does not include a commitment to developing CMH care, which could contribute to bridging the health gap between Uzbekistan and Western Europe^13^.

The aim of this paper is to provide evidence-based recommendations for the reform of the Uzbekistan's mental health care. This policy report provides (1) a description of the current mental health system in Uzbekistan, and (2) a discussion of reforms in mental health systems of EE countries. Based on these findings, recommendations for deinstitutionalization in Uzbekistan are proposed.

## Methods

### Review of Uzbekistan's mental health system

A literature search on the Uzbekistan's mental health system published between 1991 (year of Uzbekistan's independence) and April 2019 was conducted across four databases (Medline, Embase, PsychINFO and Web of Science). The search strategy (appendix) was based on a previous review of mental health systems in EE^14^.

Literature on the epidemiology of severe mental disorders (ICD-10 diagnosis: F20–F22, F24, F25, F28–F31, F32.3, and F33.3), available mental health services and staff, policy and legislation, financing of mental health care, stigma and service users' involvement was included. Severe mental disorders were selected due to their high socio-economic burden^15^. The inclusion criteria were extended to include opinion papers, reports and editorials. All literature published in English or Russian languages were included. Clinical, biological, psychometric research papers, case studies, conference abstracts were excluded. After title and abstract screening, studies that did not meet inclusion criteria were excluded as not relevant and the rest were screened for full-text. Identified studies were complemented with World Health Organization (WHO) reports (e.g. Health Systems in Transition, WHO-AIMS and Mental Health Atlas). State websites (e.g. lex.uz and minzdrav.uz) were used to obtain documents on mental health law, legal acts and presidential decrees concerning mental health. Data from included articles were coded and extracted according to topics mentioned above. A narrative synthesis was used to summarize the information.

### The impact of mental health reforms on deinstitutionalization in Eastern Europe

Mental health care transition from institutes to CMH in EE was evaluated through psychiatric beds reduction and availability of community services. Mental Health Atlas^16^–^19^ was used for psychiatric beds change calculation and availability of community mental health services was analyzed through 23 country profiles in the Lancet's review of mental health systems in EE^20^. Georgia, Lithuania and Poland were identified as countries which had achieved the most progress in the transition to CMH in EE for their significant inpatient bed reduction and commitment to scale-up community services. Details with tables on the process of identification of these three countries can be found in the appendix.

Selected countries were then further examined by literature referenced in the corresponding country profiles in the Lancet review^14^. Key milestones in the development of mental health policies and systems were extracted for each of the identified countries as a particular focus on the factors that contributed to reform and CMH and challenges experienced. Activities, milestones and barriers common across identified countries were explored. Triangulation of information identified through the literature review was used to develop a set of recommendations for mental health reform in Uzbekistan.

## Results

### Mental health care in Uzbekistan

**Figure 1.**
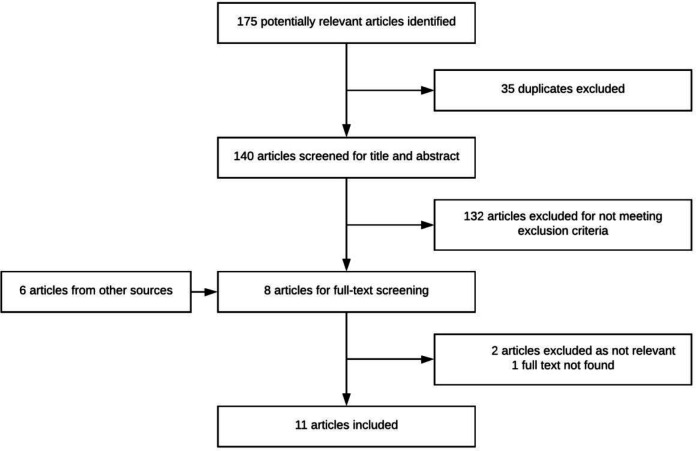
Screening and selection of articles

One hundred and forty unique records were identified through the search strategy (Figure 1). Following title and abstract screening, eight full-text articles were reviewed. Two articles did not meet inclusion criteria and one article could not be found. An additional three legislation and policy documents and three national reports were included.

Uzbekistan adopted its first mental health law, *On Psychiatric Care*, in the year 2000, which ensures the rights of people with mental disorders and outlines involuntary hospitalization procedures^21^. In July 2018, a draft of the amended law was published for public consultation. This included more thorough regulation of involuntary hospitalization, including court appeal process and state supervision over the activities of psychiatric care provision^22^. A national mental health policy and plan, which focused on shifting services from hospitals to the community was also introduced in 2000. However, it was not accompanied by an increase in funding and remains largely unenacted^23^. A number of legal acts were published in 2013-2014. These aimed to improve psychiatric provision through incentivizing mental health workers and ameliorating patient quality of life^24^.

The proportion of mental health expenditure has varied and declined since independence. The latest available data from 2012 reports 2.1% of the total health budget is spent on mental health^25^. Additional funds have been allocated to mental health care since 2013 by the Ministry of Finance together with loans and grants from foreign states for the reconstruction of psychiatric facilities and purchase of medical equipment^12^,^25^.

Before independence, primary care was neglected in favor of secondary and tertiary care provision^26^. This was and still is particularly true for mental health. Most (89%) of the mental health budget is spent on psychiatric hospitals while integration of mental health into primary care remains poor^23^,^27^. Outpatient care is provided by 22 psychiatric dispensaries located across the country, including day treatment in some regions^28^.

Although psychiatric hospitals continue to receive the majority of the mental health budget, the number of beds in mental hospitals and psychiatric dispensaries has declined nearly two-fold since 1991^28^. However, the associated savings from reduced bed numbers were not reallocated to create community-based services but, instead, diverted to other health care concerns^28^. Today, the number of beds in psychiatric hospitals is 26 per 100,000 population but it does not fully reflect the institutionalization of people with mental disorders in Uzbekistan. An additional 890 beds are located in forensic inpatient departments and 6,500 beds for people with mental disabilities across 20 social care homes^26^. In addition, tens of thousands of children with special education needs are placed in more than 200 special boarding schools in the country^25^. It is not clear whether reduction of psychiatric beds is associated with transinstitutionalization of people from hospitals to other institutions since information on these facilities is not publicly available.

Due to the stigmatization of the field of psychiatry, relatively low salaries and poor working conditions, the number of psychiatrists working in Uzbekistan has declined nearly two-fold since 1991 to 2.8 per 100,000 population^25^. However, the country retains a high number of psychiatric nurses, with nine per 100,000. Social workers and psychologists are not integrated into the health system. There are only 0.08 psychologists per 100,000 population and no social workers or occupational therapists involved in mental health care^17^. To address the lack of human resources for mental health, the 2018 presidential decree identified increasing access to psychiatric care as a priority and promotes the inclusion of social workers within the mental health care system^12^. Quotas for postgraduate studies and medical residencies in psychiatry have increased in each medical school and scholarships are now provided.

Stigma associated with mental disorders within the country has been related to a decrease in help-seeking from medical professionals and greater demand for traditional healers^26^,^29^. Mundt et al.^11^ suggest this finding is partly a result of the integration of Muslim spiritual and Russian biological traditions in Uzbekistan's psychiatry.

### Mental health care development in Georgia, Lithuania and Poland

Apart from showing tangible commitment to the provision of mental health in the community by developing services integrated into state health system, Georgia, Lithuania and Poland have substantially downsized inpatient capacities and provide the best examples of CMH care in the region. In Lithuania and Poland, a number of consecutive policy documents developed in the 1990's onwards set the direction of a new mental health policy^30^,^31^. New community services began as a result, but these were sporadic and largely unavailable. New strategies in 2007, in Lithuania, and in 2010, in Poland, supported the development of networks of nation-wide CMH centers to provide care for those who would have previously been cared for in hospitals. Unfortunately, they never provided an appropriate quality of care, largely lacking psychosocial elements^20^,^32^. Reform failure can be explained by low prioritization of mental health among policy-makers, which led to the underfinancing of reform programs^31^,^33^. The inability to ensure appropriate resourcing of CMH care was experienced in both countries. Poland was unable to address financial challenges during reform. New community services were poorly integrated and found themselves in competition with hospitals where the majority of funds were still allocated and used for their renovation. Reforms also lacked sufficient monitoring mechanisms to assess the efficacy and quality of implementation activities. Low prioritization of mental health in Georgia and Lithuania was partly associated with prevalent societal stigma towards mental illness, which discourages policy-makers from implementing plans to make services for people with mental disorders closer to communities^33^.

Another barrier for new community services to operate is significant lack of qualified staff that could deliver psychosocial services^20^,^32^,^34^. Under financial assistance from EU Funds Lithuania built a network of community facilities but faced with absence of specialists who could deliver quality psychosocial help, instead treatment remained pharmacological. Reform implementation is also aggravated by bureaucratic challenges of interagency cooperation between different government structures like Ministry of Health and Ministry of Social Welfare that are both responsible for new program implementation^20^.

Georgia, which was devastated by civil war in the early 1990's, presents a slightly different case of mental health care development. After change of government in 2004, a window of opportunity for reforms was opened^35^. It took several years of preparatory work to provide policymakers with evidence on poor quality of psychiatric care available within the country and cost-effectiveness of psychosocial interventions prior to adoption of the mental health reform in 2010^35^,^36^. Investment for mental health gradually increased and plans and programs were supported by foreign experts. Deinstitutionalization within the country was comprehensive including not only establishment of services in the community such as crisis teams and residential facilities (this is referred by Georgian experts as a “real” deinstitutionalization, emphasizing that the process is not just about the reduction of psychiatric beds), but also investing into capacity building and education^20^,^35^. Modern psychiatric literature was translated into the Georgian language and treatment guidelines were updated. Mental health workers received evidence-based training and a mental health research center was established. Georgian national NGOs were directly involved in mental health reform by piloting psychosocial interventions that were later scaled-up to the state level and participating in development of policy and legislation as stakeholders. Despite significant investment into CMH care, the reforms did not affect the psychiatric hospitals, which still consume most of the mental health budget (69% in 2014) and remain in poor condition with treatment being purely pharmacological^37^, emphasizing, again, importance of addressing dual financial burden during reform implementation. Table 1 presents a summary of the findings in three countries.

**Table 1.** Community mental health development in Poland, Lithuania, and Georgia

	Poland	Lithuania	Georgia
Strong and weak characteristics of CMH	Day clinics Centers for mental health Substantial reduction (40%) of beds in psychiatric hospitals Availability and organization of services is very poor	115 Multidisciplinary mental health teams in primary care clinics 40 Day-care centers opened Substantial reduction (40%) of beds in psychiatric hospitals Multidisciplinary teams are not utilized for people with SMI Drug treatment prevails over the psychosocial treatment Lack of social workers and psychologists in the teams	One of the largest psychiatric hospitals closed and beds relocated into general hospitals New residential facilities opened in a number of towns Crises teams started functioning across country Mental health education reformed Inpatient care deteriorated and treatment became more aggressive in order to discharge patients quickly to comply with new rules.
Policy and/or plans and their outcomes in terms of access to community care	Mental Health Protection Act (1994) Mental Health Programme (1994)-Access to psychiatric care worsened National Programme on Mental Health 2011-2015 -Care mostly provided in 51 large psychiatric hospitals with unsatisfactory conditions -Centers for mental health do not meet criteria of the Programme	State Programme on the Prevention of Mental Disorders 1999-2009 Mental Health Strategy (2007)-Investments took place into hospital and pharmaceutical care-Deinstitutionalization and destigmatization targets were not prioritized-Community care remained underfunded	National Health Care Strategy 2011-2015 The National Strategy and Action Plan for 2015-2020-Care mostly relies on psychiatric hospitals with unsatisfactory conditions
Key facilitating factors	User organizations participate in policy formulating and organization of psychiatric care	EU Structural Funds assisted in opening new day-care centers	State funding for mental health substantially increased after 2004 New funding model (2008) Evidence of positive outcomes of CMH provided to policy-makers Civil society involvement is substantial International donors financial support
Key Obstacles	Major health reform that changed financial scheme of health care and diluted responsibility of mental health policy Lack of funding Inadequate funding of community services Shortage of mental health staff	No mechanisms to assess efficacy and quality of implementation were in place Lack of political will Economic constrains Stigma among general population Passive user movement Shortage of mental health staff Lack of funding	Lack of state funding Shortage of mental health staff

## Discussion

Most countries in EE have made failed attempts to introduce nation-wide CMH care. Most patients continue to be treated in psychiatric hospitals despite adoption of mental health policies and implementation plans focused on deinstitutionalization. Commitments to reform mental health care remain primarily on paper. Path dependency and historical institutionalism theories help to explain these phenomena^38^. For policymakers it is extremely difficult for a radically different approach such as CMH care to be introduced, when the previous system had been developing for decades with different priorities and political meaning^7^. Petrea suggests that it is likely that availability of often ambitious plans and policies might be merely symbolic gestures to comply with international norms with little intention of enforcing them^39^. Significant societal mental health stigma, insufficient funding and scarce human resources make further challenges the reform process.

Mental health care in Uzbekistan shares many of the same characteristics as other EE countries at the start of their mental health reforms. While each country's attempts at deinstitutionalization have resulted in mixed outcomes, their experiences provide valuable information on the reasons why they failed to radically change the system despite ambitious plans and steady reduction of psychiatric beds.

Based on the experience of other countries in the region and the history of mental health care within Uzbekistan, we recommend the following actions to increase the success of continuing mental health reform:

1. *Embed financial planning for reform implementation*. Lack of funding is the primary obstacle every country faces when CMH care is attempted. Uzbekistan's average allocation of the health budget to mental health is considerably lower than in EE, which is itself twice lower than in Western Europe^9^. Lack of investment in mental health services during the 1990s led to the deterioration psychiatric hospitals and much of the current funding is spent on their maintenance, leaving little for CMH to develop. Based on the experiences of Georgia, Lithuania, Poland and Uzbekistan, as well as the global experience of deinstitutionalization, it is vital that adequate funds are made available to support mental health care in the community and psychiatric hospitals at the beginning of reforms with the majority of financial allocations shifting to community-based care as its coverage and treatment capabilities increase and reliance on psychiatric hospitals decreases. One can expect that after substantial economic reforms in the country and market liberalization^10^, Uzbekistan will have more financial capacity to increase expenditure on mental health care in the near future. Collaboration with international donors will also likely increase, which as in the cases of Georgia and Lithuania, will play an important role in scaling-up CMH services. However, it is important that increases in funding are thoughtfully spent in line with best evidence and existing resources. A review of the local context, strengths, needs and capacities of Uzbekistan should precede the planning and implementation of any reforms^40^. Financial planning will also address the dual financial burden of funding the old and new systems during the transitional period.

2. *Address knowledge gap and increase research capacity to assess reforms*. There is a significant gap in research on the impact of mental health reform across EE^14^. It is a particular problem for Uzbekistan as our study has highlighted the substantial lack of published literature on Uzbekistan's mental health care within international journals. A mental health research body, which does not exist currently in Uzbekistan, should be established to obtain local evidence to support implementation plans and disseminate implementation outcomes. A standardized data collection system should be in place to assist policymaking and advocacy. Failing to introduce robust assessment mechanisms and lack of prioritization contributed to the failure of Lithuanian reforms. This issue, however, was addressed in Georgia, where extensive training and educational support was made available for local professionals to challenge the old psychiatric model and an entire research center was established to support reforms and their implementation. Collaboration with international institutions will be crucial to the introduction of contemporary approaches to scientific research and strengthening of research capacity within the country.

3. *Reduce stigma associated with mental health*. High stigma towards mental illness is prevalent among general population and policy-makers across EE and is related to a long period of institutionalization of mental health^7^. In Lithuania, the authorities, although aware of deinstitutionalization policy and the notion of liberalization of mental health, were unwilling to introduce changes because of their concerns about losing the voters^33^. To avoid this in Uzbekistan, the overriding institutional culture should be challenged by national anti-stigma campaigns along with or prior to shifting care to community. Such campaigns based on local educational initiatives aiming at replacing myths and stereotypes together with mass-media advertising have reported positive outcomes both in increasing public knowledge on mental illness and in diminishing experienced discrimination reported by people with mental disorders in England, Japan, New Zealand, Egypt and Brazil^41^.

4. *Create favorable conditions for civil society groups to evolve and take part in development of mental health agenda as an equal stakeholder*. If civil society, which is comprised of charities, NGOs, service user associations and other groups united by the same goal, is not considered as a competent stakeholder, mental health care reforms are not likely to progress^42^. This is a particular problem for Uzbekistan, where our study was unable to identify a single NGO working in the field of mental health. In contrast, civil society in EE is active and raises concerns about mental health system on the political level. The lack of visibility of civil society groups within Uzbekistan may be partly due to excessively bureaucratic and opaque politics with little openness and trancparency^43^. These shortcomings have contributed to the suppression of civil initiatives and limited international aid involvement in healthcare^44^,^45^. The current Uzbekistan government, which has demonstrated openness to new ideas and reform, should start with creating favorable conditions for NGOs to evolve and international aid organizations to work in the country. Furthermore, civil society should be accepted as an equal stakeholder by the government to improve national mental health outcomes^46^.

5. *Improve the country's human resources for mental health*. Scarcity of mental health professionals is a global challenge. No strategies used by the studied countries were identified to tackle this issue. Significant efforts are needed in health workforce policy, education and financing to address the problem of labor migration and stigma among medical community to prevent the shortage to worsen^47^. In other low and middle-income countries, strategies including task-shifting and integration of mental health care with general health care already proved to be effective to compensate limited human resources^47^. Uzbekistan has addressed this issue by investing into psychiatric education and introducing new specialties such as social worker and occupational therapist. To capitalize further on the government's actions, Uzbekistan should define where these specialists will be based and in what form they will operate (e.g. multidisciplinary teams, primary care) and develop national training plans. Given Uzbekistan's cultural context, engaging traditional healers in mental health care delivery^48^ is another strategy to address this issue.

This study was initiated in light of increasing attention towards mental health care in Uzbekistan and drew on knowledge transfer from experience of other countries in the similar political context. Based on revealed obstacles and facilitators during reforms in three countries a set of recommendation was developed for Uzbekistan's policymakers. To the best of our knowledge, no such work has been conducted before.

It was not possible to evaluate the quality of policies and community services. Countries were chosen on the basis of the reported availability of mental health policies and access to community services, but not necessarily having a quality care. This is a major subject to study further in the future.

Mental Health Atlas was used as a primary evidence for the assessment of a country's psychiatric hospital capacities because it is the most complete source of information on national mental health systems currently available. Because of different approaches to collect data in different years and a large number of missing data, change only in psychiatric beds in psychiatric and general hospitals was analyzed to assess progress towards CMH. This could potentially impact the results as beds in community settings such as in residential facilities were not taken into account.

Since the initial analysis for this study was conducted, country profiles for the WHO MHA 2017 have been made available. There are no significant deviations from the data obtained from previous years' reports, except for Estonia, which reduced psychiatric bed capacity by ten-fold and increased the number of beds in general hospitals by 2.8 per 100,000 since 2005. Future studies should evaluate Estonia's mental health policies and CMH services and their success.

Uzbekistan is standing on the same path of mental health system development with other EE countries. The country was closed from external influence and had never introduced community care before. Today, when it is on the verge of reforms, it is very likely that Uzbekistan will experience the same obstacles that other countries faced with: low prioritization of mental health and persistent shortage of finances, resistance to change from medical community and general population, knowledge gap, lack of mental health and social care staff, passive civil society – these are all barriers that hinder development of community care in EE. With the change of political power, a window of opportunity in Uzbekistan was opened, where reforms started to take place and it is important to address these issues now to avoid challenges other countries had to experience. Through the creation of a financial plan, reducing the local mental health knowledge gap, tackling stigma associated with mental disorders, supporting civil society and increasing the numbers of mental health professionals, we believe Uzbekistan can give itself the best chance to find success through deinstitutionalization.
